# Autolysin mediated adherence of *Staphylococcus aureus* with Fibronectin, Gelatin and Heparin

**DOI:** 10.1016/j.ijbiomac.2018.01.047

**Published:** 2018-04-15

**Authors:** Chandni Porayath, Maneesha K Suresh, Raja Biswas, Bipin G. Nair, Nandita Mishra, Sanjay Pal

**Affiliations:** a School of Biotechnology, Amrita Vishwa Vidyapeetham, Kollam 690525, Kerala, India; b Centre for Nanosciences and Molecular Medicine, Amrita Institute of Medical Sciences, Amrita Vishwa Vidyapeetham, Cochin 682041, Kerala, India

**Keywords:** Autolysin, *Staphylococcus aureus*, Heparin, Gelatin

## Abstract

Major autolysin (Atl) of *Staphylococcus aureus*is a cell surface associated peptidoglycan hydrolase with amidase and glucosaminidase domains. Atl enzymes (amidase and glucosaminidase) are known to participate in biofilm formation and also can bind with host matrices. Earlier studies demonstrated the binding of Atlwithfibronectin, thrombospondin 1, vitronectin and heat shock cognate protein Hsc70. Here, we have shown, Atl mediates attachment of *S.aureus* to heparin and gelatine as well. The *atl* mutant strain demonstrated around 2.5 fold decreased adherence with fibronectin, gelatin and heparin coated microtiter plates. The microscopic studies confirmed the reduced binding of *atl* mutant with them compared to its parental wild type and complemented mutant strains. Amidase and glucosaminidase were expressed as *N*-terminal histidine tagged proteins from *Escherichia coli*, purified and refolded. We found refolded amidase bind with fibronectin, gelatin and heparin; whereas refolded glucosaminidase binds with only fibronectin and heparin but not gelatin. These results reemphasize Atl as one of the crucial proteins from *Staphylococcus* that facilitate their binding with multiple host cellular components during colonization and infection.

## 1 Introduction

Bacterial infections is a global concern due to its associated morbidity and mortality [1]. The first step in any bacterial infection is the contact between bacteria and the host. Bacteria interact with the host using their surface proteins and adhesins. This interaction facilitates bacterial attachment and colonization which is a requirement for their successful pathogenesis [2]. The surface proteins of streptococci are known to initiate the interaction with the serum and extracellular matrix components (ECM) leading to their colonization and disease conditions like pharyngitis, endocarditis, meningitisetc [3]. Bacterial host protein interaction also allows the Gram negative bacteria to inject their effector proteins into the host. *Shigella infection* is caused by the effector proteins injected into the host after it interacts with the host [4]. M protein of *Streptococcus pyogenes* [5], laminin binding protein (hlp) of *Mycobacterium leprae* [[6], [7]], adhesion intimin of *Escherichia coli* [[8], [9], [10]], InlA and InlB, internalins of *Listeria monocytogenes* [4] are few other examples of bacterial proteins involved in attachment to host proteins.

*S. aureus* and *S. epidermidis* are human pathogens that causes infections such as sepsis, endocarditis and implant associated biofilm infections [[11], [12]].Most of these disease conditions warrant colonization to be an important factor for successful staphylococcal pathogenesis [13]. *S. aureus* and *S. epidermidis* possess a plethora of surface proteins. These surface associated proteins play major role in their pathogenesis through their remarkable ability to bind to the host proteins. Collectively these proteins were initially known as microbial surface components recognizing adhesive matrix molecules (MSCRAMMs). It is reported that fibronectin binding protein (FnBPs) [[14], [15]], clumping factors (ClfA and ClfB) and the collagen adhesin (Cna) of *S. aureus* binds to the fibronectin, fibrinogen and collagen respectively. The SdrG of *S. epidermidis* has function similar to Cna. Studies have established the binding of serine–aspartate repeat proteins SdrC, SdrD, SdrE and bone sialo-binding protein (Bbp) of *S. aureus* and SdrF from *S. epidermidis* to collagen IV. Major autolysin (Atl) [[16], [17]] and biofilm-associated protein (Bap) [18] also are reported to mediate staphylococcal attachment to the host extracellular and plasma proteins.

Major autolysin (Atl) is one of the well-known surface protein from *S. aureus* and *S. epidermidis* [16]. Atl is a peptidoglycan hydrolase involved in bacterial cell wall degradation and cell separation during cell division [19]. *atl* gene produces a bifunctional protein (137 kDa) with amidase (AM) and glucosaminidase (GM) domains separated by three direct repeats R_1_, R_2_ and R_3_ [20]. The precursor protein is proteolytically cleaved to produce 62 kDa AM and 51 kDa GM proteins [[17], [21]]. Atl of *S. aureus* exhibits high homogeneity to the 148 kDa Atl of *S. epidermidis* [[22], [23], [24]]. Both proteins are reported to promote attachment to polystyrene surfaces and play important role in biofilm development [[16], [25]]. Additionally Atl of *S. epidermidis* also demonstrated vitronectin-binding activity which indicates itsbinding with the plasma proteins [25]. Houston et al. provided evidence to the binding of Atl repeat sequences (R_1_, R_2_ and R_3_) to the various host extracellular proteins such as fibronectin (Fn) and vitronectin (Vn) [17].

The major host extracellular proteins targeted by *S. aureus* for attachment include (Fn), fibrinogen (Fg), heparin (He) and (Vn). Fn and Fg are large glycoprotein found in body fluids, on the surfaces of cells and in the extracellular matrix (ECM) [[26], [27]]. He is an anti coagulant found in the tissue surrounding the capillaries. It is used in central venous catheters to prevent thrombosis. Studies report that *S. aureus* can incorporate heparin into the biofilm matrix and heparin coating enhances the biofilm capacity in many *S. aureus* strains [28]. Collagen is the most abundant family of structural proteins in human with wide structural variations and very often insoluble in aqueous buffer. Hence gelatin, partially hydrolysed water-soluble form of collagen, is widely used for binding assays. As described earlier, Cna of *S. aureus* binds collagen and is observed in cases of ocular infections, arthritis and osteomyelitis [[29], [30], [31]].

The *atl* gene products are directly involved in *S. aureus* pathogenicity with its diverse functions such as its attachment to polystyrene surface,lysis mediated biofilm formation and secretion of the cytoplasmic proteins from the staphylococcal cell wall [32]. Different functions of atlA protein have been elucidated and the contribution of the individual Atl protein (AM and GM) have been studied. Both the proteins have demonstrated clear role in the lysis mediated biofilm formation [33]. This article focuses on the binding property of AM and GM with the human extracellular and plasma proteins to understand their role in staphylococcal implant associated infections.

Here we have studied the role of Atl mediated attachment of *S. aureus* with Fn, He and Ge. Using an *atlA* mutant we demonstrated that AM protein interactions with Fn, He and Ge; whereas GM exhibited binding with FN and He only. Results of our study once more highlight the importance of AM and GM protein in staphylococcal pathogenesis.

## 2 Materials and methods

### 2.1 Bacterial strains

*E. coli* strains M15 (Qiagen) and Top10 were grown in autoclaved Luria-Bertani broth and the *S. aureus* strain SA113, *ΔatlA* deletion mutant and *ΔatlA* complemented strain (*ΔatlA*: pTX*atlA*) [34] were cultured in autoclaved Tryptic soy broth (TSB). In liquid culture, all bacterial strains were cultured aerobically at 37 °C with 120 rpm shaking.

### 2.2 Cloning of *am* and *gm*

The cloning and expression of *am* and *gm* gene were carried out as described previously [[20], [34]]. AM protein was expressed as *N*-terminal His-tag fusion protein from plasmid pQE30 using *E. coli* M15 as expression strain [34]. GM was expressed as *N*-terminal His-tag fusion protein from plasmid pBAD(B) using *E. coli* Top10 (pBAD-GM) as expression strain [20].

### 2.3 Expression and purification of AM and GM

The recombinant M15 *E. coli* strains carrying pQE30 plasmids with AM gene were grown to an optical density of 0.5 at 600 nm and protein expression was induced with 1 mM isopropyl β-d-1-thiogalactopyranoside (IPTG). Recombinant *E. coli* Top10 cells with plasmid pBAD-GM were induced with 0.2% arabinose. After 6 h incubation at 37° C, the cells were pelleted and the cells were lysed under denaturing condition using 8 M urea and proteins purified using Ni-NTA His bind resin (Novagen) as recommended by the manufacturer [16]. Protein refolding was carried out by adding the pooled eluted fractions of AM and GM drop by drop into the refolding buffer (0.5 mM reduced glutathione: 0.5 mM oxidized glutathione (1:1) in 50 mM Tris-HCl (pH 8.0). The ratio of protein sample to refolding buffer (volume/volume) was 1:6 to 1:8 and was kept at 4° C for 24 h with magnetic stir. The refolded proteins were further dialysed against 50 mM Tris-HCl (pH 7.4). The buffer was changed within every 3–4 h interval. The final urea concentration was diluted to 0.6 nM from 8 M. The purity of the expressed proteins were analysed by SDS-PAGE.

### 2.4 Cell (bacterial) binding assay (ELISA)

Microtitre plate wells were coated individually with Fn (0.2 mg/ml), He (0.025 mg/ml) and Ge (0.2 mg/ml) in 10 mM carbonate buffer (pH-9.2) for 4 h at 4 °C. 200 μl of wild, *atlA* mutant and complemented strains at an optical density (OD) of 0.5 at 600 nm, were added into the coated wells. The unattached cells were removed by washing the wells with phosphate-buffered saline (PBS) after 1 h incubation at 37 °C. The attached cells were fixed with 4% formaldehyde for 30 min, washed and stained with crystal violet for 10 min. The excess crystal violet stain was removed by washing with PBS. The absorbed stain was extracted using 70% isopropanol and absorbance was read at 590 nm in Synergy microplate reader using Gen5.2.05 software.

### 2.5 Microscopy

For microscopy study, cover glasses were coated individually with Fn (0.2 mg/ml), He (0.025 mg/ml) and Ge (0.2 mg/ml) in 10 mM carbonate buffer (pH 9.2) for 4 h at 4 °C. 100 μl cultures of SA113, *atlA* mutant and complement mutant strains adjusted to an OD of 0.5 at 600 nm were placed on the coverslips and incubated at 37 °C for 30 min. The non-adhering bacteria were removed by subjecting the cover glasses to PBS wash. The cover glasses were observed under microscope (Olympus BX51).

### 2.6 Interaction studies

**(i) AM and GM to Fn:** Mini analytical column with 50 μl Ni-NTA resin was prepared in a 1 ml micropipette tip sealing their exit using cotton plugs or glass wool. 50 μl of purified AM (300 μg/ml) or GM (200 μg/ml) was loaded onto the column. The column was then loaded with 100 μl of purified Fn (1 mg/ml) and the unbound fraction was collected and 25 μl of Ni-NTA agarose conjugate was added to columns. The bound proteins were eluted with 250 mM imidazole containing elution buffer (50 mM NaH_2_PO_4_, 300 mM NaCl). The eluted fractions were run on 12% SDS-PAGE to check the binding of Fn to AM.

**(ii) AM and GM to He and Ge:** The binding of the AM and GM proteins to He and Ge was tested using He and Ge agarose affinity column chromatography. The columns were prepared with 50 μl of Heparin and Gelatin at the concentration 400–1500 μg/ml and 3 mg/ml of resin. Purified AM (300 μg/ml) or GM (200 μg/ml) proteins (50 μl) were loaded onto the columns and protein was then eluted with 50 mM Tris-HCl buffer (pH 7.4) with increasing concentrations of NaCl ranging from 200 mM to 500 mM from the heparin columns and with wash buffer containing 4 M urea from gelatin columns.The eluted proteins were analyzed on 12% SDS-PAGE to check the binding.

## 3 Results and discussion

### 3.1 Decreased binding of *atl* mutant strain toFn, He and Ge

**Fig 1:**
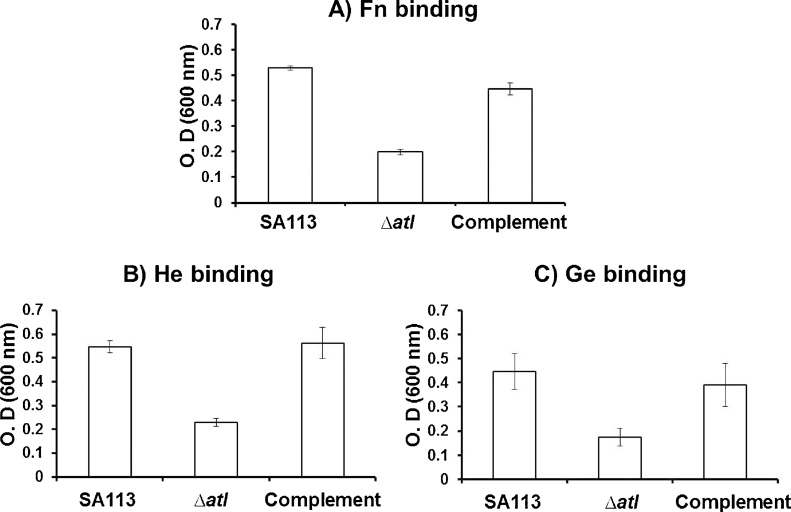
Binding of *S. aureus* wild type SA113, *atl* mutant and the complemented mutant to Fn (A), He (B) and Ge (C). *atl* mutant exhibits around 2.5 fold lesser binding to Fn, He and Ge compared to the wild type strain. The binding of complement mutant to Fn, He and Ge was similar to that of the wild type.

**Fig. 2:**
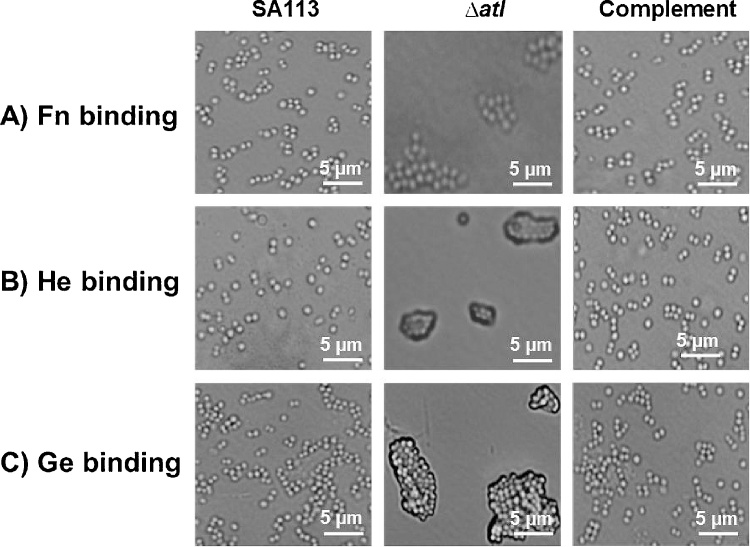
Binding of *S. aureus* strains to Fn (A), He (B) and Ge (C) visualized by microscopy. *atl* mutant strain exhibits decreased binding to Fn, He and Ge compared to the wild type SA113 and complemented strain.

Binding assays was carried out to evaluate the attachment of the wild type SA113, *atl* mutant and complement mutant (SA113pTX*atl*) strains to Fn, He and Gein 96 well plates. It was observed from absorbance values that *atl* mutant strain binds less to Fn, He and Ge compared to the wild type and complement mutant strain (Fig. 1). The binding of *atl* mutant was 2.5 fold lesser than the wild type SA113 strain. As expected, the binding of complemented mutant with Fn, He and Ge was almost equivalent to that of the wild type SA113 strain. Result of the binding assays lead to the hypothesis that *atl* gene products play major role in the binding of *S. aureus* to the human extracellular proteins Fn, He and Ge. Further, the increased binding of SA113, *atl* mutant and complemented mutant with Fn, He and Ge were microscopically visualized (Fig. 2). Light microscopic images demonstrated the reduced binding of *atl* mutant with Fn, He and Ge than its parental SA113 strain and the complemented mutant (SA113pTX*atl*) strain. The imaging studies further substantiate the importance of *atl* gene in staphylococcus adherence to human ECM.

### 3.2 Differential binding of AM and GM with Fn, He and Ge

**Fig. 3:**
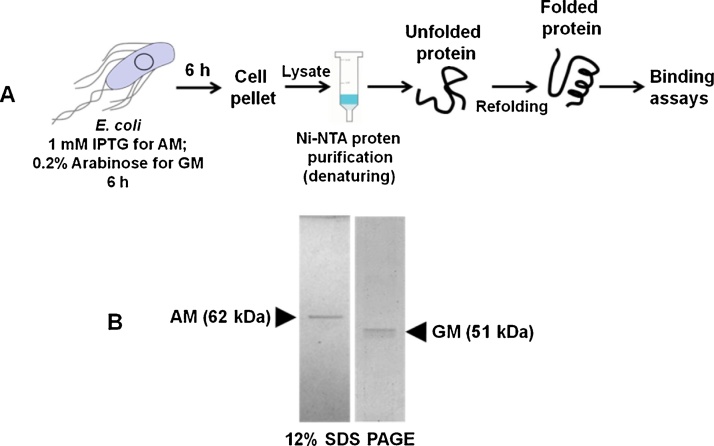
(A) Schematic representation of steps involved in protein purification and refolding. (B) 12% SDS gel image of the purified recombinant AM and GM proteins. GM is 51 kDa.

As the binding of the *atl* mutant was significantly less with Fn, He and Ge, we next studied the binding of the purified AM and GM with the Fn, He and Ge. AM and GM proteins were purified using Ni-NTA affinity chromatography under denaturing condition and the purified proteins were refolded as described in the material and methods section. The purity of the AM and GM proteins were analysed by 12% SDS-PAGE (Fig. 3).

**Fig. 4:**
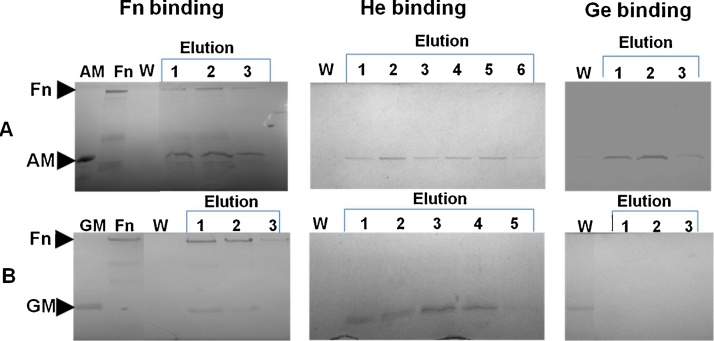
Differential binding of AM (A) and GM (B) with Fn, He and Ge analysed on 12% SDS-PAGE. AM binds with Fn, He and Ge whereas GM shows binding affinity to Fn and He only.

The binding of 6X histidine tagged AM and GM proteins with Fn, He and Ge were assessed by affinity chromatography. Fn, He or Ge was coupled with the chromatographic stationary matrix as described above. The AM and GM proteins were allowed to interact with Fn, He or Ge and eluted out as described earlier. The eluted AM and GM protein fractions were analysed on 12% SDS PAGE to understand their binding affinityforFn, He or Ge (Fig. 4). The absence of AM and GM proteins in the wash fractions and their presence in the elution fractions indicated both protein binds with Fn and He. However, chromatographic assays demonstrated no significant binding of GM with Ge. The entire GM protein loaded onto the Geagarose column was seen to appear in the wash fraction and absent in the eluted fraction indicating GM protein has non-significant affinity for Ge.

Atl sequence is highly conserved among different Staphylococcal species [35]. Previous studies have demonstrated that AtlE from *Staphylococcus epidermidis* binds with matricellular proteins thrombospondin-1 (hTSP-1), vitronectin (Vn) and heat shock cognate 71 kDa protein (Hsc70) [[36], [37]]; autolysinAas from *S. saprophyticus* [38] and AtlC from *S. caprae* binds with Fn [39]. In this report, we provide evidence that AM domain of Atl binds with Fn, He and Ge; and the GM domain binds with Fn and He.

## 4 Conclusion

Both *S. aureus* and *S. epidermidis* produces a number of surface proteins which serve as virulence factors. Atl is a well-known cell surface associated multifunctional protein from staphylococcus species. The Atl autolysin is not only involved in cleaving the PG layer, but also in interacting with human extracellular proteins [Vn, Fn, matricellular protein thrombospondin 1 (hTSP-1)], plasma proteins and polystyrene surfaces. Its role in exoprotein secretion and biofilm development has recently been elucidated. We were able to confirm from our binding assays that Atl has a significant role in staphylococcal attachment to Fn, He and Ge as the attachment was 2.5 fold lesser in the *atl* mutant strain compared to the wild type while the complement mutant strain showed binding ability similar to the wild type. Our results also affirm that the AM domain of the Atl is the major contributor in staphylococcal pathogenesis as it binds to Fn, He and Ge while GM binds to Fn and He and not Ge. Overall, we conclude that interaction of Atl protein with Fn, Ge and He facilitates host colonization of this bacteria.
